# The Effects of Prenatal Diet on Calf Performance and Perspectives for Fetal Programming Studies: A Meta-Analytical Investigation

**DOI:** 10.3390/ani12162145

**Published:** 2022-08-21

**Authors:** Sandra de Sousa Barcelos, Karolina Batista Nascimento, Tadeu Eder da Silva, Rafael Mezzomo, Kaliandra Souza Alves, Márcio de Souza Duarte, Mateus Pies Gionbelli

**Affiliations:** 1 Department of Animal Science, Universidade Federal Rural da Amazônia, Parauapebas, PA 68515-000, Brazil; 2 Department of Animal Science, Universidade Federal de Lavras, Lavras, MG 37200-900, Brazil; 3 Department of Animal and Dairy Sciences, University of Wisconsin-Madison, Madison, WI 53706, USA; 4 Department of Animal Biosciences, University of Guelph, Guelph, ON N1G 2W1, Canada

**Keywords:** fetal programming, maternal nutrition, protein requirements, systematic review

## Abstract

**Simple Summary:**

Prenatal nutrition can reshape an animal’s developmental trajectory, with persistent long-term consequences for the progeny. In prenatal life, the effects induced by the intrauterine conditions can be expressed as changes in offspring growth and meat quality. Thus, there are diverse sources of variations that contribute to the variations reported for fetal-programming responses in beef cattle, making it difficult to interpret and apply the results. In this sense, finding a common factor among the studies with which to group them may offer an opportunity to quantify the fetal-programming effects holistically and to obtain more applicable responses. With the increasing number of publications, it is important to summarize the quantitative measurements available in the scientific literature. In the present study, data from 35 publications were used. We verified that there is a gap related to the effects of maternal nutrition to females, at the beginning of gestation, and in zebu and crossbred animals, indicating new perspectives for future fetal-programming studies. In summary, our findings highlight an association between prenatal energy and protein supply and its effects on the offspring birth weight, weaning weight, and the daily weight gain of pregnant beef cows during pregnancy.

**Abstract:**

This meta-analysis aimed to identify knowledge gaps in the scientific literature on future fetal-programming studies and to investigate the factors that determine the performance of beef cows and their offspring. A dataset composed of 35 publications was used. The prenatal diet, body weight (BW), average daily gain (ADG) during pregnancy, and calf sex were elicited as possible modulators of the beef cows and their offspring performance. Then, the correlations between these variables and the outcomes of interest were investigated. A mixed multiple linear regression procedure was used to evaluate the relationships between the responses and all the possible explanatory variables. A knowledge gap was observed in studies focused on zebu animals, with respect to the offspring sex and the consequences of prenatal nutrition in early pregnancy. The absence of studies considering the possible effects promoted by the interactions between the different stressors’ sources during pregnancy was also detected. A regression analysis showed that prenatal diets with higher levels of protein improved the ADG of pregnant beef cows and that heavier cows give birth to heavier calves. Variations in the BW at weaning were related to the BW at birth and calf sex. Therefore, this research reinforces the importance of monitoring the prenatal nutrition of beef cows.

## 1. Introduction

The growth and carcass characteristics of beef cattle are mainly considered to be dependent on genetics and all of the management practices (environmental conditions) that the animals are exposed to in their postnatal lives [1,2]. However, an additional factor, which consists of prenatal nutrition, can also reshape the animal development trajectory and cause persistent long-term consequences—a concept known as fetal programming [3]. Maternal nutrition affects the nutrient partitioning [3] and acts as a signal-promoting epigenetic modification [4], which causes alterations in gene expression [5,6,7]. Thereafter, in the postnatal life, these effects induced by the intrauterine conditions may be expressed as alterations in the offspring growth [8,9], in the relative masses of muscle and adipose tissue (lean-to-fat ratio) [10], and the meat yield [11] and quality [12]. Therefore, a broader understanding regarding the effects of maternal dietetic manipulations during gestation on the offspring is crucial to produce herds with the desired quality.

It is widely accepted that both undernutrition and overfeeding may program the characteristics of offspring in utero [13,14]. However, each response is dependent on the dietary level, (i.e., the extent to which the requirements are being met by the maternal diet), the length of the application of the maternal nutritional plan, and the period of embryonic or fetal development when the treatments were applied. Therefore, there are diverse sources of variation that contribute to the variability reported for fetal-programming responses in beef cattle production, making it difficult to interpret and apply the reported responses. In this sense, the discovery of a common factor among these studies with which to group them may provide an opportunity to quantify the fetal-programming effects holistically and to obtain more applicable responses. Moreover, with the increasing number of publications, it is important to summarize the quantitative measurements available in the scientific literature. This strategy may be an opportunity to clarify some conflicting concepts using integrative research. For these purposes, a systematic review associated with a meta-analysis is a powerful tool to aggregate results from a variety of studies, potentially providing new findings and indicating new paths to perform further studies [15].

Therefore, this study aimed to (1) provide information regarding the factors that may affect offspring performance and the beef cows’ ADG during pregnancy, and (2) identify the knowledge gaps in the scientific literature involving fetal-programming studies of beef cattle, indicating new paths for further efforts.

## 2. Materials and Methods

### 2.1. Study Selection Criteria and Relevance Screening

The present study was performed through a systematic review. As data collection was performed using the available literature, the approval of the ethics committee was not necessary.

The scientific literature search was done through PubMed^®^, Science Direct, Google Scholar, and Web of Science platforms. The production animals focused on were pregnant beef cows and/or heifers and their offspring, including males and/or females. The nutritional plan of interest consisted of the information regarding the meeting of the energy and protein requirements promoted by each maternal nutritional plan applied during gestation. The studies were not selected for the application of maternal treatment in a specific window of prenatal development. Thus, studies involving the application of maternal treatment during early (0–100 days of gestation), mid (100–200 days of gestation), and/or late gestation (200 days of gestation to parturition) were considered.

**Figure 1:**
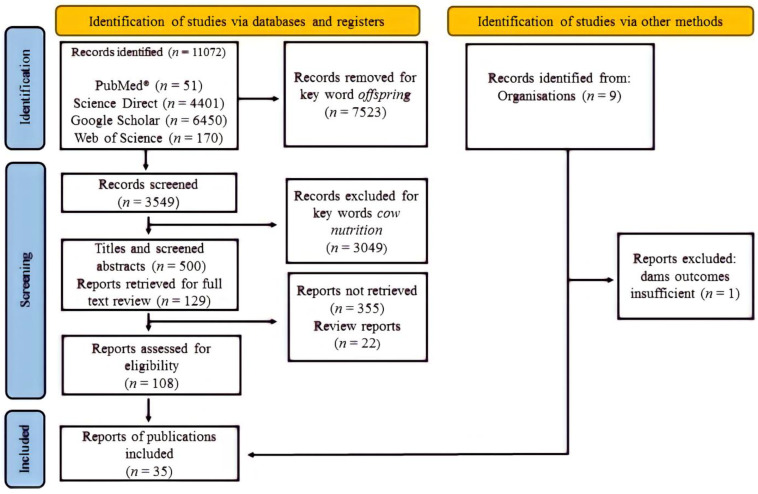
PRISMA flow chart of literature research and study selection process at different stages of the review process.

The flow chart of the research selection process over different steps of the review course, elaborated according to Page et al. (2021) [16], is described in Figure 1. To obtain a list of eligible publications, the initial keywords used were fetal programming beef, offspring, and cow nutrition. From four electronic sources, the bibliographic research generated a total pool of 11,072 references. From these studies, 3549 remained within the analysis after the application of the filter. A total of 500 were selected based on title and abstract. After reading these studies’ abstracts, 129 studies were saved and fully read. From these studies, 108 were elicited within the list of possible outcomes of interest. Nine studies were removed due to the absence of input data from cows arising from a lack of clarity regarding the experimental design, diet composition, period (early, mid, or late gestation) and duration of treatments application, or absence of initial and final cows’ BWs. 

An exploratory data analysis was performed to identify the main outcomes published in the fetal programming papers and thus elicit the target outcome of this meta-analysis. In this sense, more than 40 variables were identified in the studies, such as offspring performance, meat-quality traits, blood parameters, and gene expressions, among others (Figure S1: interest outcomes over studies used in the exploratory step of meta-analysis). After this step, considering the quantity of data availability in selected publications, the offspring performance during the cows’ calf phase was defined as the target outcome of interest for this meta-analysis. Thus, 72 publications without this focus were removed from the dataset. After the screening, a new file was formed in an Excel spreadsheet using the selected papers. Moreover, to improve the dataset regarding zebu beef cattle information, the pool in the present research included studies derived from master’s and Ph.D theses that met the described criteria or those derived from studies in progress, using information kindly provided by researchers. 

Finally, the dataset was composed of 35 publications, comprising a pool of 3854 animals, carried out in the USA (17), Brazil (14), Australia (2), and Argentina (2). A brief characterization of the publications is shown in Table 1, Table 2 and Table 3. 

### 2.2. Process of Data Extraction

After the screening, information extracted was stratified as (1) manuscript-related information (authors; year; link of publication and country where research was conducted), (2) as general information (breed of animal used; the number of experimental units; system utilized; detailed description of treatments); (3) as maternal information (all data available from the pre-natal period), and as (4) outcome information. For each outcome, the number of animals per treatment, mean, standard error of the mean, other variability measures (i.e., standard deviation or the coefficient of variation), and the *p*-values were registered. In studies in which the exact days related to the length of gestation were not stated (*n* = 9), they were considered as 285 days and 292 for *Bos taurus* and *Bos indicus*, respectively [48].

Some of the studies (*n* = 20) used in the dataset did not provide information regarding the meeting of dam-metabolizable energy (ME; Mcal/day) and metabolizable protein (MP; g/day) requirements during treatment application. When this information was not available in the articles, but the authors provided enough information to estimate ME and MP requirements, the requirements were calculated according to the nutritional requirements system referenced in the study. To compare the nutrient supply among diets, the levels of ME (Mcal/kg) and MP (g/kg) intake during the treatment application were separately calculated as requirement percentages. The energy and protein supply of 13 studies performed using taurine or crossbred beef cattle were estimated according to BCNRM (2016), while in 7 studies the requirements were achieved according to the Nutrient Requirements of Zebu and Crossbred (BR—CORTE 3.0) [49].

### 2.3. Data Analysis

Firstly, an exploratory analysis considering the Pearson correlation coefficients using the quantitative variables of our dataset was performed. After analyzing the correlations, to study the relationships of possible explanatory variables with the response variables of interest, a mixed multiple linear regression procedure was used considering the random effects of studies [50]. Studies were considered as random effects in the statistical model because there was no interest in exploring the causes of heterogeneity among studies [51]. First, a complete model was established contemplating all possible response variables of interest. The random effect of the studies was considered on the regression parameters and the significance of these effects was checked by using the likelihood-ratio test. To select the fixed effects from the full model, the stepwise backward method was used, which was based on the significance of each variable identified in the F test of the analysis of variances (α = 0.10). Once the model was selected, multicollinearity verification was performed by investigating the variance inflation factors (VIF) of each variable, with highly collinear variables being considered those with a VIF greater than 10, as recommended by Kaps et al. [52]. Additionally, the following statistics were extracted from the full and reduced models: Akaike criterion [53] and residual standard deviation. Conditioned and marginal r-squared coefficients for the reduced model were calculated as suggested by Nakagawa et al. [54]. Marginal r-squared describes the proportion of variance explained by the fixed effects alone [σ²fixed/(σ²fixed + σ²random + σ²residual)]. Conditional r-squared describes the proportion of variance explained by both fixed and random effects [(σ²fixed + σ²random)/(σ²fixed + σ²random + σ²residual)]. 

For all analyses, the R Core Team software version 4.0 (R Core Team, Vienna, Austria) was used. The packages used for the visualizations and calculations given the correlations and hypothesis tests were ggplot2 and ggally, respectively. For model adjustment and hypothesis test, the packages lme4 and lmerTest were used, respectively. The denominator’s degrees of freedom for the fixed effects were calculated by using the Kenward–Roger method and type 3 sums of squares for the analysis of variance. The random effects were estimated using the residual maximum likelihood method. The criterion used to weigh the observations was the number of animals that composed each variable mean measured in each publication.

Moreover, we explored the interaction between ME and MP supply for dam over gestation and offspring birth BW. For the calculations, 500 kg was considered as the average body weight of pregnant cows [55].

## 3. Results

### 3.1. Dataset Characterization

**Figure 2:**
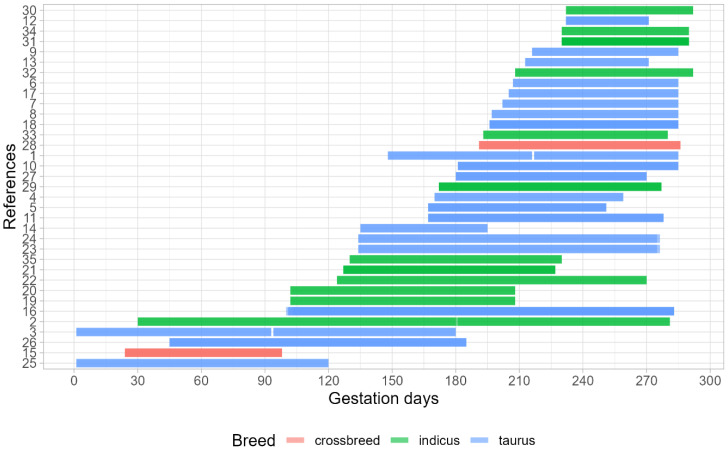
Summary of gestation period and breeds for all publications that composed the dataset to perform this meta-analysis.

The descriptive statistics of the data used for the statistical analysis are described in Table 4. The summarization of the randomized controlled studies used, according to the gestational period of treatment application and the cow breed, is described in Figure 2. It is noticeable that ~94% (33 from 35 publications) of the publications used in the present meta-analysis involving the gestational nutrition effects on the offspring performance were focused on mid and/or late pregnancy. Thus, a scarcity of publications that were focused on gestational nutrition during early gestation was identified. In addition, 60% of the publications (21 from 35 publications) evaluated the effects on males and female offspring in the same study, while 40% focused only on males (14 from 35 publications). Taurine animals were used in 61% of the studies (blue bars in Figure 2), zebu animals in 31% of studies (green bars in Figure 2), while crossbreeds (red bars in Figure 2) comprised 8% of the studies. Thus, the presence of only two studies contemplating cross-bred animals made their use in our analyses unfeasible.

### 3.2. Correlations between Explanatory Variables and Interest Outcomes

**Figure 3:**
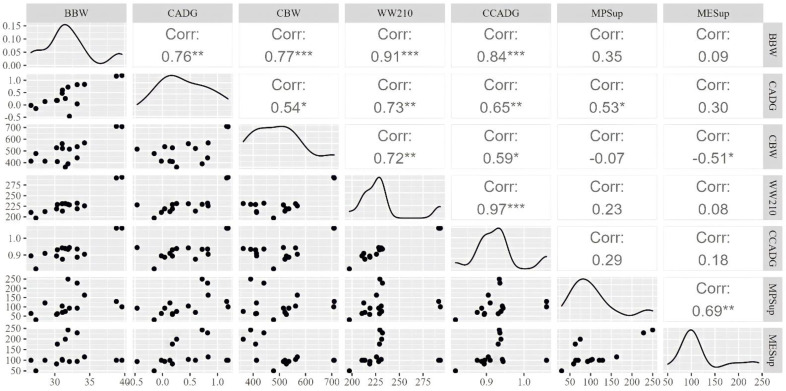
Main diagonal: densities of all continuous variables from the dataset (BBW = birth body weight, kg; CADG = cow average daily gain, kg/d; CBW = cow body weight, kg; WW210 = weaning weight adjusted to 210 d, kg; CCADG = average daily gain cow-calf phase, kg/d; MPSup = metabolizable protein supply, %; MESup = metabolizable energy supply, %). Values above the main diagonal: Pearson correlation coefficients among the variables used, where * = *p* < 0.05, ** = *p* < 0.01, and *** = *p* < 0.001. Scatter plots below main diagonal: graphical representation of relationships among all variables.

The significant correlations of cows’ ADG and cow’s mean BW during pregnancy, calf weaning weight (adjusted to 210 days), calf’s ADG at cow-calf phase, ME, and MP supply were verified (*p* < 0.05; Figure 3). The cow’s mean BW during pregnancy was positively correlated with the calf’s birth body weight (r = 0.67; *p* < 0.001), the calf’s weaning weight (r = 0.52; *p* < 0.001), and the calf’s ADG at the cow-calf phase (r = 0.46; *p* < 0.01). A strong relationship (r = 0.70; *p* < 0.001) between the calf’s ADG at cow-calf phase and weaning weight was observed. The weaning weight was related to the MP supply of the dam during pregnancy (r = 0.40; *p* < 0.05). The MP and ME densities present in Figure 3 (main diagonal) demonstrated that the MP and ME supply presented frequencies of distributions with similar behaviors in the present dataset. In the same way, there was also a positive relationship between the MP and ME supply during pregnancy (r = 0.57; *p* < 0.001).

### 3.3. Predictor Variables of Cows’ ADG over Pregnancy and of Offspring Performance

After correlation analysis, the stepwise regression included the following data as predictor variables of cows’ ADG and of offspring performance: the cows’ mean BW during pregnancy, cows’ ADG, the ME and MP supply during gestation, cows’ breed, productive system, calf’s birth weight, and the offspring sex. Of the possible variables of the full model, the variables that were retained, that is, those that were statistically significant and that did not present collinearity issues (VIF less than 10), were included in each model. 

Based on this, the regression analysis showed a positive association between the level of MP supply and the cows’ mean BW during pregnancy with the cows’ ADG, as described in Equation (1) (Table 5). In this sense, prenatal nutritional planes with higher protein levels seem to improve the cows’ ADG. Furthermore, improving the cows’ mean BW during pregnancy seems to be effective for enhancing the cows’ ADG during pregnancy. In Equation (2), the percentage of protein and energy requirements met, as well as the cows’ BW, was used as a calf birth BW predictor (Table 5). The regression model demonstrated that heavier pregnant beef cows and cows under high nutritional planes, in general, give birth to heavier calves.

Considering the conditional r-squared, the selected model was responsible for substantial amounts of variation in the calf birth weight, which was equivalent to 53%. In contrast, using the marginal r-squared model, the model’s explicability for the calf birth weight was reduced to 21%. There was a similarity of the values found for the Akaike information criterion (AIC) between the full and reduced model regarding the calf birth BW outcome, and for all other the outcomes of interest. Moreover, despite the proximity of the AIC values, in the reduced models for all outcomes, these values were lower. 

Equation (3) suggests that calf birth weight and maternal ME supply during gestation would be associated with the calf’s ADG during the cow-calf phase (Table 5). Concerning the calf’s BW at weaning, our model explains about 51% of the variation in this characteristic, which was influenced by the calf’s sex, birth weight, and the cow’s energy supply, as described in Equation (4). 

Overall, the residual standard deviation (RSD) values between the full and reduced model were very close for all outcomes.

**Figure 4:**
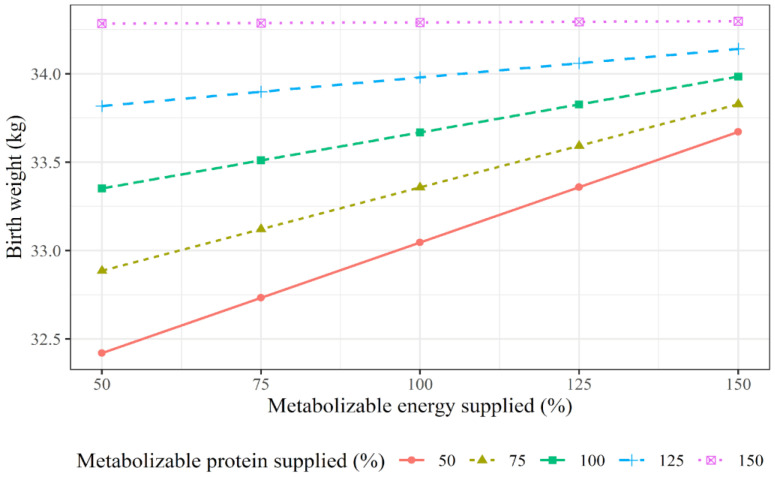
Effect of levels of supplying both energy and protein requirements of a 500 kg cow from mid- to-late gestation on calving birth weight.

The interaction between the ME and MP supply for dam during the gestation and offspring birth BW is described in Figure 4. A higher energy and protein supply resulted in a greater calf birth BW. However, the magnitude of the response in birth BW becomes more discreet when the protein and energy supply for the dams are excessive.

## 4. Discussion

### 4.1. Factors That Can Affect the ADG of Beef Cows during Pregnancy 

The model’s ability to explain all the outcomes of interest was lower when using the marginal r-squared than when using the conditional r-squared. This suggests that the impact of the experiment’s effect was very high in the present meta-analysis, which is justified by the presence of our heterogeneous dataset. Due to this, the explanation of the data variability by our potential model was diluted in relation to the other effects that could not be quantified and included in the analyses.

In this sense, the regression approach was used to understand how the target interventions may change the outcomes’ behaviors and in what direction this pattern occurs. This is because although there were studies focused on different stages of pregnancy within the dataset, there was an unequal distribution related to the application of maternal treatments across the studies. Thus, this assumption was required to avoid potential confounding effects [56]. Nevertheless, the compression of the interrelationships among the management practices during pregnancy, with respect to the gestational physiology, and in the offspring traits at the postnatal phase is indispensable for making integrated and assertive decisions about the production system of livestock. The regression analysis showed that the nutritional strategies employed during gestation that increase the metabolizable protein supply [such as protein supplementation programs for females fed poor-quality forage using crude protein- (CP), rumen-degradable protein- (RDP), or rumen-undegradable protein- (RUP) based supplements, as well as the use of rumen-protected functional amino acids, may lead to improvements in the pregnant beef cows’ ADG. Notably, since the MP and ME supply for the dams presented a positive relationship and similar behaviors related to the frequency of distributions in the present dataset, it is possible to infer that both energy and protein levels are important for manipulating cows’ ADG. 

In the late pregnancy, when most studies used in this meta-analysis were concentrated, the bovine fetuses presented an accelerated growth [57], which resulted in greater glucose and amino acid (AA) demands to attend to the fetus’s anabolism and its oxidative metabolism [58]. Consistent with this, there is evidence that pregnant beef cows receiving a low-protein diet tended to have a higher circulating pool of total AA and a greater glycogenic AA blood concentration than pregnant cows fed with protein supplementation, indicating the greater skeletal muscle catabolism to attend the fetal demand in un-supplemented beef cows [40]. This pattern was associated with a lower ADG for cows under a low-protein nutritional plan. Therefore, increasing the dams’ MP supply through the diet is an effective way to reduce the amplitude of maternal tissue mobilization during pregnancy, improving the cow’s ADG. 

In addition, these findings demonstrate the importance of gestational nutrition towards the beef cows’ longevity. Maternal nutrition planes during gestation promote carryover effects on the beef cows’ reproduction indexes in the subsequent breeding season, on their milk/colostrum yield [59], and on the milk/colostrum composition of their subsequent lactation [60], affecting the financial viability of a cow-calf enterprise. Reproduction, for example, is strongly influenced by the cows’ nutritional status upon calving [47], which in turn is affected by the nutritional strategies employed during pregnancy. The literature has shown that cows in moderate body conditions at calving have greater reproductive indexes than cows in thin or obese conditions [61,62,63]. This is explained by the hypothalamic–pituitary axis suppression in undernourished cows [64,65]. Thus, if animals lose body weight and if adequate stores of fat are not available, hypothalamic and pituitary hormone (such as gonadotropin-releasing hormone (GnRH), luteinizing hormone (LH), and follicle-stimulating hormone (FSH) releases will be suppressed, and estrous cycles will be not initiated. In contrast, the available evidence [66] indicates that an excessive body score condition during pregnancy may increase the embryonic losses in cattle, impairing the reproductive indexes in beef cattle operations.

The maternal prenatal nutrition may also affect the subsequent lactation of beef cows. Prenatal nutrition may impair lactogenesis through endocrine modifications that cause a colostrum production delay at the end of the gestational period in ruminants [67]. In mammals, the onset of lactogenesis is triggered by a decrease in progesterone levels and by an increase in estrogen and hydrocortisone concentrations, which together induce the synthesis of prolactin, which in turn promotes α-lactalbumin synthesis [68]. α-lactalbumin is a rate-limiting enzyme for lactose synthesis, the main osmotic agent in the mammary secretory epithelium [69]. Accordingly, Banchero et al. [67] found that undernourished ewes (fed 70% of their daily energetic requirements) had lower colostrum accumulations than their contemporaries (fed 110% of their daily energetic requirements). Impaired colostrum synthesis is associated with a delay in the rate of progesterone withdrawal levels before parturition [59], lower prolactin levels, decreased insulin and IGF1 levels (related to the mammary gland differentiation and development) [67], and reduced mammary gland weight [60] in undernourished females. Moreover, females with a better body condition score may present a greater contribution of bodily reserves to supply the udders’ demand for the synthesis of milk and its components [70]. Likewise, the available evidence studying first parity ewes showed lower total solids-not-fat, lactose, protein, and urea N in the milk of pregnant females exposed to feeding restrictions (60% of their requirements) compared to their contemporaries fed with 100 and 140% of their nutritional requirements [71].

### 4.2. Maternal Aspects That May Affect the Offspring Performance Later in Life

The regression model that was generated showed that heavier cows, in general, calved heavier calves. In our study, the breed was not retained in the final model due to the potential confounding with the cow weight. Furthermore, this pattern was confirmed by the differences in the BW means of taurine (570.0 ± 81.9) and zebu (500.0 ± 45.2) beef cows’ weights in our study. This finding indicates that the diversity of breed groups in cow-calf systems may cause phenotypic differences related to calves’ birth weights. Thus, following these findings, it is expected that taurine breeds (such as angus cows) produce heavier calves than zebu or crossbreed beef cows [72]. 

Not only genetic but also environmental factors may affect a calf’s birth BW [73]. The investigation of energy × protein supply in cows from the mid-to-late gestation (Figure 4) demonstrated that both significantly impact the calves’ birth BW. Previous reports related that energy levels in late pregnancy seem to have a more accentuated influence on this variable [74]. Our analysis showed that lower energy and protein levels for pregnant beef cows reduce the calves’ birth BW. This occurs because the shortage of nutrients impairs the placental delivery of nutrients to fetuses [75], which could reduce the pregnant tissues’ accretion (fetus, placental, uterus, and amniotic fluids) and the gestational growth, leading to a reduced calf-birth BW [14]. These reports support our findings, in which low nutritional plans were related to lower calf birth weights, demonstrating the importance of monitoring the prenatal nutrition of pregnant cows to fully meet the nutritional requirements of the dams. 

In addition, the response pattern verified in Figure 4 demonstrates that when excessive levels of energy and protein are used, the magnitude of the response in the calf birth BW to maternal nutrition becomes more discrete. Indeed, although over-nutrition may bring some benefits such as increased offspring marbling [76], concerning the birth weight, this practice seems to represent a futile supply of nutrients and energy. This is in turn explained by the lower magnitude of the responses in the offspring’s weight gain, which became more discrete in response to the exacerbated nutrient availability (even though the exact point at which this happens has not been determined).

The negative effects of an inadequate prenatal diet may be persistent throughout the offspring’s postnatal life, impairing the hypertrophy of skeletal muscle at the cow-calf phase [25]. Herein, a reduced weaning weight may be associated with poor maternal nutrition during pregnancy [77]. Muscle growth during the postnatal life in cattle occurs by the existing muscle fibers’ hypertrophy, which in turn occurs through the support of nuclei donation from satellite cells [78]. Previous studies demonstrated that nutritional insults from mid to late gestation may reduce the myonuclei and muscle DNA of ovine fetal muscle through effects on satellite cells’ proliferation and incorporation, impairing fetal muscle growth [79]. Evaluating the transcriptional profile of skeletal muscle, Carvalho et al. [7] found 187 and 123 genes down- and up-regulated, respectively, in response to maternal protein supplementation during mid-gestation. In summary, the molecular analysis that was performed showed favorable responses in the offspring hypertrophic processes promoted by the maternal protein supplementation in the mid-gestation phase [7].Therefore, this available evidence is consistent with the responses verified in the present study.

In addition, the lower potential for offspring development in the postnatal phase may be the consequence of the gestational-nutrition effects on their muscle fiber population. Previous reports using ruminants showed that nutritional restriction during pregnancy could impair myoblasts’ proliferation [57], reducing the number of muscle fibers through mechanisms related to changes in molecular levels [80]. Myogenesis during the pre-natal stage is a crucial event because there is no new muscle-fiber formation during the postnatal stage [81]. The formation of muscle fibers occurs in two events called primary and secondary myogenesis. Primary myogenesis is concentrated between the conception and the second month of gestation, while secondary myogenesis (when the majority of fibers are formed) occurs between the second and seventh months of gestation [3]. Myogenesis is controlled by the myogenic regulatory factors’ (MRFs) expressions, including the Myogenic Factor 5 (*MyF5*), Myogenic Differentiation 1 (*MyoD*), Myogenin (*MyoG*), and Myogenic regulatory factor-4 (*MRF-4*), whose expressions are in turn regulated by nutritional factors [82]. Cells positive for *MyF5*, which are highly specific to committed skeletal myoblastic cells, give rise to myoblasts, while cells negative for this MRF may create other cells types, such as adipoblasts and fibroblasts [83]. The *MyF5* and *MyoD* act on myoblast proliferation [57]; *Myogenin* regulates the formation of myotubes, acting directly on the differentiation process; while *MRF-4* seems to be more related to myotubes’ maturation [84]. In this sense, the available scientific evidence demonstrates that there is a smaller window for increasing the pool of myoblasts in animals exposed to nutritional insults during intrauterine development [85] due to an earlier fusion and differentiation of these cells, associated with changes in the MRFs expressions [57]. Therefore, as muscle mass is defined by the number (hyperplasia) and size (hypertrophy) of muscle fibers [3], the nutritional plan during gestation plays a central role in the offspring’s growth potential later in life, as demonstrated in the present study.

Thus, following the regression model generated in the present meta-analysis, it is expected that calves from well-fed cows during pregnancy present a higher ADG during the cow-calf phase and a greater weaning weight. These findings emphasize the importance of adopting nutritional strategies to attaining the pregnant cows’ requirements and to improving the economic benefits in beef cattle operations. It is noteworthy that although only maternal energy levels present a significant effect in the statistical model for the prediction of weaning weight, we can infer that both energy and protein supply may affect the offspring’s performance. To our knowledge, there are no studies available in which the increase in protein supply did not promote changes in the energy supply. In this sense, overall, the protein level in the diet automatically increases the energy supply. This condition is markedly evident in studies with ruminants fed low-quality forage, such as cattle raised in tropical pastures during the dry season (lower protein content) [25]. The lack of nitrogen in the ruminal environment impairs microbial protein synthesis, which in turn compromises the ruminal fiber degradation and passage rate, consequently reducing the feed intake [86,87]. In this sense, the use of protein supplementation corrects this condition, improving the dry matter intake and thus the nutrient intake, which increases the energy supply [14].

The regression model generated also demonstrated that variations in the weaning BWs are related to the calf birth BW and calf sex. A study [88] reported that for every 1 kg increase in the calf birth weight, the weaning BW increases 1.53 kg, confirming the close relationship between these variables. The present meta-analysis contemplates studies with males (castrated and intact males) or with both males and females. Additionally established in the scientific literature, males demonstrated to be heavier at weaning [72]. This response may be associated with the greater muscle fiber quantity in males [89], which is trigged by testosterone’s effects on the mesenchymal stem cells’ commitment [90]. Moreover, greater testosterone levels in males also promote greater anabolism for this group once the protein accretion is triggered by testosterone [91]. Testosterone stimulates the mammalian target of the rapamycin (mTORC1) signaling pathway through the insulin-like growth factor (IGF) [92], favoring the myofibrillar protein synthesis.

### 4.3. Knowledge Gaps in the Scientific Literature Involving Studies concerning Gestational Nutrition of Beef Cattle

The knowledge gaps in the studies focused on gestational nutrition and fetal programming approaches in the scientific literature were verified in the present study. Our findings demonstrated that in most of the studies available, treatments were applied from mid to late gestation. Consistently, Zago et al. [9] also verified the low availability of fetal programming and gestational nutrition studies focused on the first trimester of pregnancy. Herein, studies that contemplate the effects of maternal nutrition during early gestation were further explored. Nevertheless, it is important to highlight that the uterine environment is sensitive to nutritional status [93]. Thus, the scarcity of publications in the first trimester of pregnancy can be partly explained by the higher embryonic losses, possibility associated with severe nutritional restrictions applied to the dams, impairing the pregnancy success. Therefore, considering this condition, when working with nutritional insults in early pregnancy, researchers need to be prepared for a possible reduction in experimental units, which can make the logistics of work more difficult and expensive. 

In early pregnancy, the energy and protein requirements for the gravid uterus are discreet [49]. Quantitatively, the energy and metabolizable protein requirements for pregnancy are considered significant (in practical terms) from 135 days of gestation at the BR-CORTE system [49], when they represent more than 10% of the maintenance requirements. Thus, the amount of MP and ME required in this stage is small compared to the total requirements. Nevertheless, this is a critical stage because several key processes occur during early gestation. Most of the placental growth [94], as well as the uteroplacental vascular beds’ development—which is essential to support the accelerated fetal growth in late pregnancy [95]—occurs in the first half of gestation. Regarding embryonic/fetal development, several events occur in early pregnancy, such as neural tube formation [96]; the establishment of the circulatory system, including the heart, arteries, veins, and blood [97]; and fetal organogenesis [98,99]. In this sense, studies focusing on this gestation period may also provide answers about the modulation of offspring characteristics in the long term. Therefore, despite the challenges of working with gestational nutrition in early pregnancy, we encourage researchers to explore this scientific gap.

Since most of the studies utilized in this meta-analysis were related to the effects of maternal nutrition on the offspring’s performance, the preference for the second and/or third trimester of gestation for treatment applications by the authors can be possibly explained by the fetal growth dynamics. In animal production, skeletal muscle is responsible for meat production. According to the theoretical window of skeletal muscle development proposed by Du et al. [6], secondary myogenesis occurs during mid-gestation, forming most muscle fibers (as discussed above). Thus, this period seems to be the most effective period to potentiate muscle fiber formation through maternal nutrition [25]. In contrast, most muscle hypertrophy occurs in the last trimester of gestation [6], when the conceptus presents an accelerated growth. Intramuscular adipogenesis and fibrogenesis [83] are also more pronounced during the late period of pregnancy. Based on this, this window of prenatal development may be used as a strategy to enhance the calf birth weight and the quality grade of meat, which explains why most efforts are focused on the last trimester of pregnancy.

Furthermore, the majority of studies considered (which in turn were concentrated during mid-to-late gestation) were focused on the nutrient-restriction effects, while fewer studies were focused on the effects of overfeeding. Indeed, overnutrition during late pregnancy has been associated with a higher incidence of dystocia [100], which can disrupt experimental conditions. We also observed a shortage of studies designated to crossbred and Zebu beef cattle, which are widely used in the livestock of tropical countries. It is well consolidated that there are physiological differences between Zebu and Taurine breeds. Bos indicus are more resilient animals. Therefore, under a challenging environment, Bos taurus breeds present a disadvantage and experience more accentuated negative consequences [101]. These differences, among others, raise questions about whether fetal-programming responses differ between taurine and zebu animals. Nevertheless, we still do not have enough information about this since it is unknown if it is possible to extrapolate the research results concerning fetal-programming studies between different breeds. So, further investigation is necessary to clarify this question, and researchers are encouraged to investigate a possible interaction between maternal nutrition and breeds in future studies [72]. 

Another noteworthy knowledge gap verified in this study was the limitation regarding studies contemplating solely female offspring. Thus, the interpretation of the effects of prenatal nutrition responses on the females’ performance can still be better solidified by future fetal-programming studies. Moreover, the responses related to the maternal nutrition plan during pregnancy occur in a sex-dependent manner and seem to be triggered by an evolutionary mechanism. According to the Trivers–Willard theory [102], the major well-known and investigated set of sex-biases hypotheses among biologists [103], the maternal investment in mammals is greater for the sex that provides greater marginal returns [104,105]. In cattle, this differential investment seems to occur in favor of females when the dams were exposed to a challenged nutritional scenario during gestation, due to their greater reproductive role compared to males, which represents a ‘‘safe bet’’ for the species’ perpetuation [106,107]. In addition, male fetuses were less energetic and expensive because they are typically larger and heavier than females [108], suggesting greater nutrient requirements for males during fetal growth. Thus, this is another factor that contributes to dams investing more resources in female fetuses when exposed to conditions of undernutrition [103]. Consistently, Ithurralde et al. [106] verified that under a potentially challenging nutritional condition, dams carrying male fetuses preserve their own condition relative to dams carrying female fetuses, which consequently results in more deleterious effects in males [21]. Evaluating uterine hemodynamic behavior, Meneses et al. [109] verified greater resistance, pulsatility indexes, and a greater systolic/diastolic ratio for beef cows carrying females, demonstrating this different pattern of maternal resource allocation between male and female fetuses. Nevertheless, little is known about which mechanisms modulate these responses, and how the dams may ‘sense’ their offspring’s fetal sex to promote sex-biased adjustments based on the environmental conditions [110]. However, this physiological and behavioral negotiation between the dams and their offspring in different species seems to occur through hormonal signals (such as stress and steroid hormones) and by bioactive molecules from a fetal origin in the maternal–fetal system [103,110,111]. 

The intra-uterine development patterns between females and males are also different, which in turn explains the different fetal-programming responses as a function of offspring’s sex later in life. Some authors [89] found differences in the intra-uterine muscular development between sexes in response to prenatal maternal nutrition, with a greater skeletal-muscle development for males than for females during the same window of development. Lastly, the epigenetic signatures also seem to occur in a sex-dependent manner [112,113], consisting of an additional factor that contributes to explaining the different response patterns between males and females in the postnatal stage. According to Nugent and Bale [114], there is a greater global placental DNA methylation and greater DNA methyltransferase (DNMT) activity for females compared to males in the placenta, as well as differences in placental autosomal gene expression at the mRNA and protein levels related to the offspring’s sex. In addition, trophoblast cells originate from the embryo, carrying its sexual chromosomes (XX or XY), which in turn promote sex-effects of the placental biochemistry, function, and signaling. The X chromosome seems to undergo less rigorous epigenetic repression (gene silencing) in response to the intrauterine conditions compared to the Y chromosome, which guarantees greater protection for females under a potentially challenging scenario [114]. Therefore, these findings highlighted that future fetal-programming studies must consider the sex of the offspring.

Furthermore, we identified a knowledge gap regarding possible associative effects between different stressor conditions that might reprogram the progeny’s developmental trajectory in the long term, such as emotional or environmental stress. To our knowledge, there are no studies demonstrating the association between the effects of maternal nutrition and thermal stress, which is a common field-verified condition. Thermal stress may cause epigenetic alterations in the offspring, thereby changing the gene expression pattern [115]. Thermal stress also affects placental function, impairing its glucose, amino acid, and oxygen transport capacities (lower substrate permeability), promoting a lower placental clearance [116]. Thus, associative effects between different stressful stimuli (i.e., unfavorable environmental conditions and dietary insults) probably accentuate the negative responses in the offspring. Therefore, this type of questioning in future research may contribute to guiding the management practices in countries where pregnant cows are exposed to this type of condition.

## 5. Conclusions

In summary, our results showed that prenatal diets with greater protein or energy levels may improve pregnant beef cows’ ADG and that heavier cows, in general, give birth to heavier calves. Furthermore, this work highlighted that there is an associative effect between the energy × protein supply and the calf birth BW and that maternal nutrition during gestation affects the offspring’s performance at the cow-calf phase. These findings reinforce the importance of monitoring the prenatal nutrition of pregnant beef cows to enhance their performance and that of their offspring, especially for pregnant cows kept under nutritionally challenging production conditions. Furthermore, this systematic review indicates that there is a gap related to the effects of maternal nutrition concerning females, concerning maternal nutrition in early pregnancy and concerning Zebu and crossbred animals, and these factors may be considered in future fetal-programming studies.

## Figures and Tables

**Table 1:** Publications (*n* = 28) using the protein level as comparative parameters.

References	Dams Breed	Description of Maternal Treatments Application during Gestational Period	Gestational Period	Feeding System	Offspring Sex
[17]	Crossbred	Fed a high- (14% CP) (1) or low- (7% CP) (2) protein diet	Early gestation	Feedlot	Male
[13]	*Bos* *taurus*	Fed with improved pasture (1) or with a native range (2)	Early and mid-gestation	Pasture	Male
[18]	*Bos* *taurus*	Fed to provide 100% of NRC requirements (1); limitedly fed to provide 70% of treatment 1 diet (2) or limitedly fed to provide 70% of treatment 1 diet plus a protein supplement to promote an essential AA supply to the small intestine equal to treatment 1 diet (3)	Early and mid-gestation	Feedlot	Mix
[11]	*Bos* *taurus*	Pasture plus a low (1) or high crude protein supplement (2)	Early and mid-gestation	Pasture	Mix
[19]	*Bos* *taurus*	Limitedly fed to provide 75% or 250% of CP requirements at early gestation or to provided 228% or 63% of CP requirements	Mid-gestation	Feedlot	Mix
[20]	*Bos* *taurus*	(1) Unsupplemented from mid to late-gestation or (2) supplemented with protein to late-gestation	Mid-gestation	Pasture	Male
[21,22]	*Bos indicus*	Fed with poor quality forage without (1) or with a CP supplement (2)	Mid-gestation	Feedlot	Mix
[23]	*Bos indicus*	Fed with poor quality forage plus nitrogenous mineral salt (1) or with supplement rich in a non-degradable rumen protein (2) or with other supplements rich in rumen-degradable protein plus ground corn (3)	Mid-gestation	Feedlot	Mix
[24]	*Bos taurus*	(1) Unsupplemented, (2) supplemented with the distiller-based supplement, or (3) supplemented with corn gluten-based supplement	Mid and late gestation	Feedlot	Mix
[25]	*Bos indicus*	(1) Unsupplemented during entire pregnancy; (2) supplemented with protein supplement from early to mid-gestation or (3) supplemented with a protein supplement at late gestation	Mid and late gestation	Pasture	Mix
[12,26]	*Bos* *taurus*	Fed with low (1) or high (2) protein diets	Mid and late gestation	Feedlot	Mix
[27]	*Bos* *taurus*	Limitedly fed to provide 102% or 80% of CP requirements during mid to late gestation or during late gestation	Mid and late gestation	Feedlot	Male
[14]	*Bos* *indicus*	Fed with poor-quality forage plus mineral salt provided ad libitum without (1) or with a crude protein supplement (2)	Mid and late gestation	Pasture	Male
[28]	*Bos* *indicus*	Fed with high- (1) or low- (2) rumen-undegraded protein	Mid and late gestation	Feedlot	Mix
[29]	*Bos* *taurus*	A cow managed under different wintering systems: grazing winter range (dormant Sandhills) vs. corn residue; within grazing treatment received or did not receive a protein supplement	Late gestation	Pasture	Male
[30]	*Bos* *taurus*	Pasture plus a 36% CP supplement provided at the level of 454 g/cow 3 times a week (1); pasture plus a self-fed supplement comprising 50% animal protein sources and 50% trace mineral package (2) or brief and intermittent supplementation using the same supplement of treatment 1	Late gestation	Pasture	Male
[31]	*Bos* *taurus*	Not supplemented (1); supplemented with 36% CP supplement provided at the level of 454 g/cow 3 times a week (2) or self-fed supplement of 28% CP supplement	Late gestation	Pasture	Male
[32]	*Bos* *taurus*	Cows managed to enter the last trimester of gestation with a low (4 points) or high (6 points) body score condition, with each group being fed without (1) and with DDGS supplementation (2)	Late gestation	Pasture	Mix
[33]	*Bos* *taurus*	Not supplemented (1) or supplemented (2) with dried distillers grains plus solubles	Late gestation	Pasture	Mix
[34]	*Bos* *taurus*	Limitedly fed (1) with corn co-products and ground cornstalks or (2) ground-mixed, cool-season grass hay to provide 62% or 113% of rumen-degraded protein, respectively	Late gestation	Feedlot	Mix
[35]	*Bos* *taurus*	Limitedly fed to provided 100% or 129% of CP requirements	Late gestation	Feedlot	Mix
[36]	*Bos* *indicus*	Fed with pasture without (1) or with a crude protein supplement (2)	Late gestation	Pasture	Mix
[37]	*Bos* *indicus*	Fed with pasture without (1) or with a crude protein supplement provided at the level of 0.5 kg/day (2), 1.0 kg/day (3), or 1.5 kg/day (4)	Late gestation	Pasture	Male
[38]	*Bos* *indicus*	Fed with pasture without (1) or with a crude protein supplement (2)	Late gestation	Pasture	Male
[39]	Crossbred	Diets provided to promote low (1), medium (2), and high (3) nutritional levels	Late gestation	Pasture	Mix
[40]	*Bos* *indicus*	Fed with pasture without (1) or with a crude protein supplement (2)	Late gestation	Pasture	Male

**Table 2:** Publications (*n* = 5) using the energy level as comparative parameters.

References	Dams Breed	Description of Maternal Treatments Application during Gestational Period	Gestational Period	Feeding System	Offspring Sex
[41]	*Bos* *taurus*	Three primary energy sources: (1) Fed ad libitum with grass hay (high-fiber concentration), (2) limitedly fed with corn (high-starch concentration), and (3) limitedly fed with dried corn distillers grains with solubles (high fiber, protein, and fat concentrations).	Mid and late gestation	Feedlot	Mix
[42]	*Bos* *taurus*	Fed for promoting a positive (1) or negative energy balance (1)	Mid and late gestation	Pasture and Feedlot	Male
[43]	*Bos* *taurus*	Limitedly fed to promote a severe restriction (50% of requirements) (1), a moderate restriction (75% of requirements) (2), or to meet 100% of requirements (3)	Mid and late gestation	Feedlot	Male
[44]	*Bos* *taurus*	Limitedly fed to provided 100% (1) or 125% of TDN requirements (2)	Late gestation	Feedlot	Mix
[45]	*Bos* *taurus*	Not supplemented (1) or fed with a bunk supplement at the level of 2.16 kg/ cow/day (2) or at 8.61 kg/cow/day (3)		Pasture	Male

**Table 3:** Publications (*n* = 2) using the protein and energy levels as comparative parameters.

References	Dams Breed	Description of Maternal Treatments Application during Gestational Period	Gestational Period	Feeding System	Offspring Sex
[46]	*Bos* *indicus*	Pasture (1); pasture plus a daily (2) or infrequent energy-protein supplementation (3)	Late gestation	Pasture	Mix
[47]	*Bos* *indicus*	Fed with pasture without (1) or with an energy-protein supplementation (2)	Late gestation	Pasture	Mix

**Table 4:** Descriptive statistics of the variables collected to compose the dataset for cows and their offspring.

Variables	*n* ¹	Number of Means ²	Minimum	Mean	Median	Maximum	SD ³
Cow							
Days on treatment	3854	88	39	95.2	89.0	251	35.2
Stage of gestation ^4^, day	3854	88	1	154	172.0	232	66.8
Metabolizable protein supplied, %	3854	88	30	109	102.0	298	44.8
Metabolizable energy supplied, %	3854	88	50	108	100.0	243	32.4
Body weight, kg	3337	75	362	523	516	710	78.5
Average daily gain, kg/d	3122	74	−0.8	0.23	0.22	1.19	0.44
Offspring							
Birth weight, kg	3121	71	26.6	35.3	35.8	44.0	3.53
Weaning weight 210 d, kg	2386	46	176	249	232	345	43.4
Weaning age, day	2462	53	82	178	185	245	39.9
Average daily gain cow-calf phase, kg/d	2182	44	0.68	0.93	0.94	1.15	0.10
Total gain cow-calf phase, kg	2386	46	142	214	200	310	41.7

^1^*n* = total number of animals. ² Number of means = number of treatments means used. ^3^ SD = standard deviation. ^4^ Stage of gestation = period of gestation when the study started.

**Table 5:** Effects of cows’ BW and diet during pregnancy on the offspring performance based on the regression analysis.

Response Variables	Candidate Variables ¹	Equation Number	Model Selected	Model Statistics			
				Full ²	Reduced ³	R-Squared ^4^	
						Conditional	Marginal
Cow ADG, kg	CBW, MPSup, MESup, MPSup × MESup, breed, and system	Equation (1)	−1.93 + 0.004 × CBW + 0.002 × MPSup	AIC ^5^: 34.3; RSD ^6^: 0.782	AIC: 33.5; RSD: 0.790	0.32	0.10
Calf birth BW, kg	CBW, CADG, MPSup, MESup, MPSup × MESup, breed, sex, and system	Equation (2)	16.09 + 0.029 × CBW + 0.025 × MPSup + 0.0187 × MESup − 0.0001 × MPSup × MESup	AIC: 207.9; RSD: 3.92	AIC: 204.6; RSD: 3.92	0.53	0.21
Cow-calf ADG, kg	BBW, CBW, CADG, MPSup, MESup, MPSup × MESup, breed, sex, and system	Equation (3)	0.623 + 0.0075 × BBW + 0.0005 × MESup	AIC: −77.1; RSD: 0.18	AIC: −83.4; RSD: 0.19	0.22	0.03
BW at Weaning 210 adj., kg	BBW, CBW, CADG, MPSup, MESup, MPSup × MESup, breed, sex, and system	Equation (4)	165.73 + 4.34 × BBW + 0.226 × MESup − 87.33 × sex	AIC: 237.2; RSD: 24.8	AIC: 235.9; RSD: 23.2	0.85	0.51

¹ Dummy variables for sex [male = 0 and mix (female + males) = 1], system (pasture = 1 and feedlot = 0), and breed (Bos indicus = 1 and Bos taurus = 0); BBW = birth body weight [minimum = 26.6 kg and maximum = 44.0 kg]; CADG = cow average daily gain [minimum = −0.833 kg and maximum = 1.192 kg]; CBW = cow body weight [Minimum = 362 kg and maximum = 709 kg]; CCADG = average daily gain cow-calf phase, kg; MPSup = metabolizable protein supply, % [Minimum = 30% and maximum = 298% of requirements]; MESup = metabolizable energy supply, % % [Minimum = 50% and maximum = 243% of requirements]. ² Full statistical model: The full statistical model includes all candidate variables that possibly affect the response variable. ³ Reduced statistical model: The reduced statistical model includes only the candidate variables that our analysis methodology defined as the most relevant. ^4^ R-squared: conditioned and marginal r-squared for the reduced model. Marginal r-squared describes the proportion of variance explained by only the fixed effects. Conditional r-squared describes the proportion of variance explained by both fixed and random effects. ^5^ AIC: Akaike information criterion (smaller is better). ^6^ RSD: Residual standard deviation.

## Data Availability

Data will be made available upon reasonable request to the corresponding author.

## Supplementary Materials

The following supporting information can be downloaded at: https://www.mdpi.com/article/10.3390/ani12162145/s1, Figure S1: Interest outcomes over studies used in the exploratory step of meta-analysis.
