# Supplementary figures and images for: The Effects of Prenatal Diet on Calf Performance and Perspectives for Fetal Programming Studies: A Meta-Analytical Investigation
Source: Animals (Basel). 2022 Aug 21;12(16):2145. doi: 10.3390/ani12162145 (PMC9404886; doi:10.3390/ani12162145)

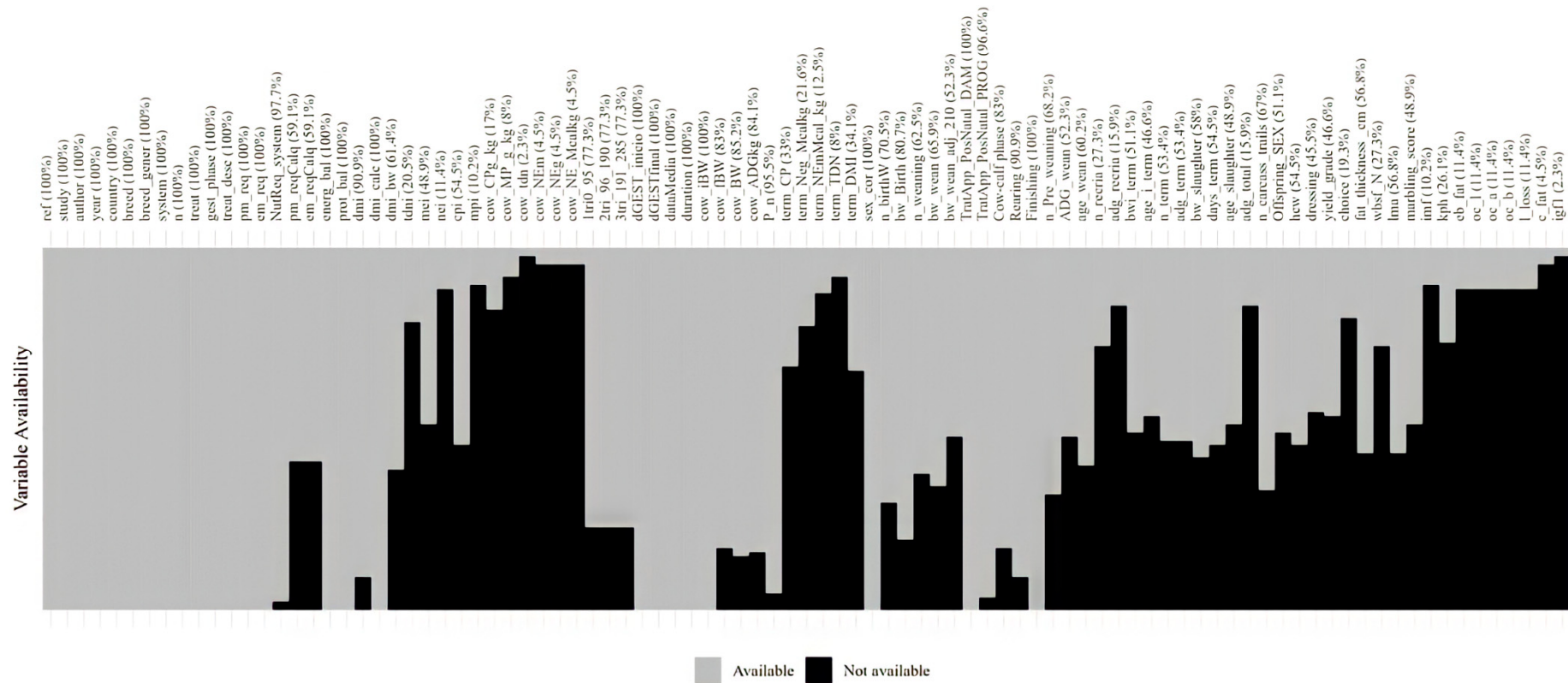

**Figure S1.** Interest outcomes over studies used in the exploratory step of meta-analysis.

Supplement: Supplementary file 1 [file animals-12-02145-s001.zip › animals-1813326-supplementary.pdf]
